# A Practical Experience on the Amazon Alexa Integration in Smart Offices

**DOI:** 10.3390/s21030734

**Published:** 2021-01-22

**Authors:** Răzvan Bogdan, Alin Tatu, Mihaela Marcella Crisan-Vida, Mircea Popa, Lăcrămioara Stoicu-Tivadar

**Affiliations:** 1 Department of Computers and Information Technology, “Politehnica” University of Timisoara, 300006 Timișoara, Romania; alin.tatu@4sh.fr (A.T.); mircea.popa@upt.ro (M.P.); 2 4SH France, 6 Rue des Satellites Bâtiment C, 33185 Le Haillan, France; 3 Department of Automation and Applied Informatics, “Politehnica” University of Timisoara, 300006 Timișoara, Romania; mihaela.vida@upt.ro (M.M.C.-V.); lacramioara.stoicu-tivadar@aut.upt.ro (L.S.-T.)

**Keywords:** voice assistant, internet-of-things, smart office, project management tool, Amazon Alexa, Jira, usability, sentiment analysis

## Abstract

Smart offices are dynamically evolving spaces meant to enhance employees’ efficiency, but also to create a healthy and proactive working environment. In a competitive business world, the challenge of providing a balance between the efficiency and wellbeing of employees may be supported with new technologies. This paper presents the work undertaken to build the architecture needed to integrate voice assistants into smart offices in order to support employees in their daily activities, like ambient control, attendance system and reporting, but also interacting with project management services used for planning, issue tracking, and reporting. Our research tries to understand what are the most accepted tasks to be performed with the help of voice assistants in a smart office environment, by analyzing the system based on task completion and sentiment analysis. For the experimental setup, different test cases were developed in order to interact with the office environment formed by specific devices, as well as with the project management tool tasks. The obtained results demonstrated that the interaction with the voice assistant is reasonable, especially for easy and moderate utterances.

## 1. Introduction

The Internet of Things (IoT) has become a technical revolution by which different kinds of machines and devices can be interconnected via Internet. Such kind of physical objects are called things and their functioning goal is to offer information about the surrounding environment and, based on external stimuli, to appropriately become reactive. Therefore, a new spectrum of services and applications has emerged due to the opportunity of interconnecting physical devices and the virtual space. One of the technological novelties where these principles are laying the ground is that of smart offices. Their role is “to integrate physical devices, human beings and computing technologies with the intention of providing a healthy, conducive, interactive and intelligent environment for employees” [1]. Such kind of places are dynamically evolving to provide a favorable environment for planning daily tasks and later on to serve the means of administering employees activities at work. In smart offices, sensors in conjunction with actuators work together in order to achieve the main goal of such systems which is enhancing employee’s efficiency [2]. Devices which are added into smart offices should support people in completing their tasks in a proactively fashion [3].

Voice assistants (VAs) have been part of the requirements of smart environments from the inception of human–computer interaction, continued later on with Ambient Intelligence [4]. Over the last few years, the emergence of voice assistants became progressively influential in our daily routines. In 2011, Apple became a pioneer in terms of intelligent voice assistants by integrating Siri into their smartphones, being initially used to conduct web searches. Since then, different other inexpensive consumer-level products have been developed. Google Assistant [5], Microsoft Cortana [6], and Amazon Alexa [7] are some of the most popular at the moment, each of which is trying to solve and automate our daily routines such as: home or office automation, ambient control, accessibility, production line automation. Moreover, open source variants that respect the same design principles are developed, such as Mycroft [8] and Jasper [9]. The interactions with voice assistants are becoming a new norm in a very short time such as asking questions about the weather, news, setting bedtime alarms, playing music, and traffic notification. It can only be assumed that voice enabled devices are more engaging and have better retention since interactions are done in an obvious manner, through natural language, as opposed to other devices such as laptops and smartphones that require certain periods of time to get accustomed and understand its functionalities. However, the task completion is the most important metric impacting user satisfaction [4]. Three factors have been defined for successful interactions with VAs: (1) contextual assistance in terms of understanding user’s location; (2) update offers by taking into account user interests; and (3) using tasks completion in order to provide further suggestions [10].

In the context of smart offices, one of the main challenges when dealing with VAs is that VAs should be capable of offering employees means of tasks operations and also execution [11]. Our research is based on the motivation and necessity to innovate but also experiment with state-of-the-art technologies, this being the reason to integrate voice assistants in a smart office in order to encompass different routines and tasks that can be performed at the workplace. This supports the research effectiveness of using VAs for smart offices. Basic facilities of such an environment is comprised of lighting, air conditioning, and an employee attendance system (regular office space setup). One important research challenge we are trying to address is to understand whether using VA integrated with project management tools and services can improve the work efficiency or not. For the scenario of our paper, we propose to interact with Jira (https://www.atlassian.com/software/jira), which is a proprietary project management tool developed by Atlassian. It provides project planning, issue tracking, release management, and reports for the business. Our work investigates the possibility to integrate third party applications, and in particular to provide speech interaction with the Jira project management tool, in order to support office tasks as reading, creating and managing issues and projects, and interacting with users in performing tasks. As the integration of VAs has offered different architectural proposals in the past years, we aim at validating the existing knowledge available from similar scientific studies, in terms of data sets, users, methodologies, test conditions and limitations, technical insights, and guidelines. The findings and results of the usability of our approach are providing new scientific insights for projects on similar characteristics, by describing the used methodologies, technical implementations, lessons learned, and limitations for certain use-cases and users’ categories.

Based on the observations noted above, the research questions in this study are as follows: (RQ1) To build the architecture needed to integrate voice assistants into smart offices in order to support employees in their daily activities, like ambient control, attendance system, and reporting, but also interacting with project management services used for planning, issue tracking, and reporting; (RQ2) to understand what are the most accepted tasks to be performed with the help of voice assistants in a smart office environment; (RQ3) to analyze our system based on task completion and sentiment analysis, aiming to offer new scientific insights to benefit researchers on further work of similar features.

The structure of the paper continues with: Section 2 presenting previous work regarding smart offices and voice assistants, followed by Section 3 containing the methodology used for our research, the technical requirements specifications of the system with practical use cases further being discussed. Next, the paper continues with the general framework for integrating a voice assistant into smart offices and the system architecture and the detailed software service architecture. The usability evaluation, the results, and discussion are presented in Section 4, while the last part of the paper is reserved for the conclusions of our research.

## 2. Previous Work

### 2.1. Smart Offices’ Implementations and Applications

Different types of approaches have been presented in the scientific literature which address smart offices as well as specific problems identified in building and optimizing such kind of systems. Authors present in [12] an application that is identifying users using facial recognition. The implementation is based on an Edge system capable of computing the increasing of image compression levels, but also on the possibility of hybridizations of Cloud and Edge computing in order to optimize computational resources. Ref. [13] presented an integrated semantic service platform that supports ontological models for IoT-based services. The personalized smart office environment results by interpreting the user’s input via a smartphone. Ref. [14] offered a solution capable of identifying a certain task and, based on that, the light from a smart bulb will be adapted accordingly. The system is also capable of predicting future activities, but also to offer reliable recommendations. In order to save more energy, authors of [15] describe a system for lighting control in an office, based on considering the sensor output as an observation of the occupancy status, while Ref. [16] presents a smart office based on a Raspberry Pi board which is able to perform different levels of security of the outside environment. The system is also capable of detecting dangerous situations occurring in the office, such as the presence of intruders, thieves, or fire. This makes it clear that cloud services are to be used for resource pooling and broad network access [17]. Different smart environment projects are based on using cloud services for offering the desired functionalities, for example in the field of a cloud based smart home environment [18], but also for managing computing and storage resources needed in the case of medical emergencies [19].

The idea of using VAs in a smart office was first introduced by authors in [20]. However, the solution presented in [20] lacks tangible results in terms of interaction with the VA. The interaction with the VAs is usually realized in a single language, but there are projects that have extended the capabilities into understanding several languages [21]. 

### 2.2. State-of-the-Art on Voice Assistants Usage and Implementation

#### 2.2.1. Voice Assistants for Medical Applications

When dealing with the research papers pertaining to VAs, it can be noted that there are different examples of using these devices in implementing medical-based applications. It is interesting to notice that Ref. [22] is presenting a study showing that currently only one-eighth of pediatric providers are using VA technology in their clinical setup, while 47.4% manifested their willingness in trying digital voice assistants. In [23], VAs are used to understand medication names in USA and Canada, but the researchers’ conclusion is that such kind of devices should not be used for the moment as a reliable source of medical information and guidance. A distinct application category is that of employing VAs to help the elderly that have visual impairment [24,25], need strengthening of social bonds [26], or daily caring [27,28]. In [29], Amazon Echo Dot was used in the home of seven older adults. This VA was consistently used for finding online health-related information, while the usage of other features, like setting timers, reminders, and so on, was low due to reliability issues. Ref. [30] presented a system used to enhance the training process and increase the performance of combat medics and medical first responders. The implemented VAs are real-time monitoring and responding to each trainee. A large number of patients are taking the role of managing their health. VAs are used in Ref. [31] in order to help patients build up their health literacy, while in Ref. [32] to assist them in managing diabetes medication. Ref. [33] illustrated the impact that VAs have on the market of health and fitness apps. Due to the restrictions VAs currently have on recording health data, but this kind of market is still mainly focused on health education and fitness due to privacy and security reasons. A special class of medical applications is that of using VAs for visually impaired people [24,25,34,35]. Amazon Alexa and Apple Siri are the two VAs used for conducting experiments in this case. While the individuals appreciated the services offered by these devices, understanding the responses and controlling the presented information were some points which further need improvement [36].

#### 2.2.2. Voice Assistants for Educational Activities

Virtual Assistants have started being used in different educational contexts. Authors in [37] present an intelligent multi-agent based voice-enabled virtual assistant developed specifically for interacting with the Moodle Learning Management System (LMS). The motivation behind developing this VAs was enhancing the usability of LMS in order to speed-up user’s tasks through voice commands. The projects in [38,39,40,41,42] show practical uses of VAs to assist engineering students into completing each stage of experiments, controlling hardware laboratory instrumentation, but also presenting supplementary teaching resources when asked by the user. Work in [43] studies the impact digital assistants have on children, as these are adapting the language style of the software when talking to real people. It presents a speech assistant called “Eliza” which is rebuking the impolite requests. The experiments in [44] show that children prefer the human to human interaction in their different activities. An interesting project where VAs have been successfully used is Cyrus [45], an application which allows test database adaption, without being limited to a specific set of keywords or natural language sentence structures. This project has two main modes: the tutor mode allows students via VA to choose an example database, accepts voice queries in natural English, and maps the query to SQL. In assessment mode, the application shows only the test queries in English and the difficulty level which was chosen by the student to transcribe in SQL. In the described prototype of the paper, the focus was to support a sufficient number of SQL query classes for an entry level database class and to allow multiple natural language renditions of the queries to support variability and personalization.

#### 2.2.3. Addressing the Security in Voice Assistants

Giving the open nature of voice assistants, one of the issues to be addressed is that of security threats [46,47]. Authors of [48] present two-proof of concept attacks, namely fake order and home burglary. The study shows that VAs should have further authentication mechanisms, as well as additional sensors in order to correctly interpret the environment. Work in [49] proposes a solution for increasing the reliability of interacting with different VAs by using intelligent agents, while Ref. [50] offers a solution for continuous authentication based on the recognition of owner’s voice. The false positive rate is less than 0.1%, while this system is capable of detecting impersonation attacks, replay attacks, and mangled voice attacks.

#### 2.2.4. Voice Assistants for Entertaining Activities

Virtual Assistants are used for entertaining purposes as highlighted in [51], where these devices are integrated into an Android application in order to control multimedia applications. Ref. [52] presented a case study of using VAs for building a driving assistant companion. This advanced driver-assistance system is offering drivers different information by predicting upcoming events on the road based on the data received from range finding sensors.

#### 2.2.5. Voice Assistants Helping the COVID-19 Crisis

Facing the worldwide COVID-19 crisis, the validity of VAs being applied in different scenarios has been tested, but also questioned. These days, the emergency facilities are many times contaminated by the deadly virus. The humanity is facing an unseen deadly enemy. Every technological device that could be used is a valuable asset in preventing the contamination with the virus. This is why VAs could be used with healthcare communication, like asking standard exam questions, triaging, screening, receiving questions for providers, and offering basic medication guidelines [53]. Such implementations would decrease dependency on providers for routine tasks, but also reduce the impact of delayed care. Ref. [53] concluded that different VAs showed disconnection with public health authorities, while the information presented by the VAs is many times not up-to-date and not reliable. This is why there is a need to improve the features of VAs, but also the coordination between the stakeholders in terms of requirements and necessities. The state-of-the-art scientific literature review shows a somehow different situation in terms of other COVID-19 affected areas. Ref. [54] presented an interesting case of integrating voice assistants into a Moodle-based Learning Management System. The students were divided into two groups, the first group participated in the online activities without any VA support, while the second one faced the interaction of VAs. It is interesting to note that greater satisfaction was found in the group in which VAs have been applied, but no better results were found in the group that used the voice assistants.

Compared to the different approaches highlighted from the state-of-the-art literature, our smart office implementation is based on an Amazon Alexa voice assistant. In Ref. [14], the tasks are being recognized with the help of a smartphone, which could be an alternative to our approach. However, the scientific trend around VAs demonstrates that different scenarios are taken into consideration to analyze the areas where these devices could be successfully used, but also which are the points where further research and development is still needed. Our decision for using VAs is based on this motivation. The main difference between the research results in [15] and our proposal is that the light in our system is controlled via the VA and not based on the occupant seat. An improved smart office concept could include both methods. However, the novelty of our proposal is that a prototype of the integration is being constructed and tangible results of this are further obtained. This is the reason for which key points of future research are identified.

Compared to the medical applications where VAs were used in the presented scenarios, before COVID-19 crisis and during it, we are facing similar results when dealing with complex scenarios, namely the tasks to be completed [36]. Our research encourages further developments into this topic, especially when the testing conditions are not lacking noise or the spoken language is not native. The satisfaction of the user is proportional to the degree that the VA is able to complete the task, this being a result of our research, as well as the research in applying VAs to education [38,39,40,41,42,54].

## 3. Method

The system described in this paper enhances a smart office with Voice Assistants, especially in the domain of project management tools interaction. The research steps our approach implemented are as follows:

1. Building the system architecture of the prototype for including the VA into a smart office environment, in order to support employees in their daily activities, like ambient control, attendance system and reporting, but also interacting with project management services used for planning, issue tracking, and reporting (Section 3.1 and Section 3.2)
2. Construction of the prototype by physically integrating the required devices and software implementation (Section 3.2 and Section 4.1)
3. Implementation of the Alexa skills for the interaction of the user with the prototype; these skills are: Jira skill, Ambient control skill, and Office skill (Section 3)
4. Performing usability evaluation (Section 4.1)
  - Performing an initial survey for the users
  - Analyzing the data in the initial survey
  - User interaction with the prototype, based on a set of experimental test cases
  - Performing a feedback survey for the users
  - Analyzing the data in the second survey
  - Validation of the results from the last question in the feedback survey with respect to the results of the first question in the feedback survey, by using sentiment analysis
  - Obtaining the polarity results of the users’ opinions
5. Analysis of the scores obtained at point 4 by using a task completion factor (Section 4.2)
  - Calculate and analyze the Kappa coefficient
6. Identify and discuss possible causes for the scores at previous points (Section 4.2 and Section 4.3)
7. Identify and discuss new scientific insights to benefit researchers with further work of similar features (Section 4.2 and Section 4.3).

The overall features our prototype offers are:

- *Attendance system:* the owner of the smart office wants to know when the employees enter and leave the office. We propose that each employee has an associated badge having integrated in it a RFID/NFC chip with which he/she can interact with the attendance sub-system. An administrator will independently add, remove, or modify new and existing users in the system.
- *Reporting:* the owner, the accountant, and manager are interested in monthly/yearly reports regarding their employee’s standings regarding their productivity and availability. For example: “How much time did John worked last week?” could be a question addressed to the VA.
- *Ambient control:* an administrator is interested to remotely control or schedule actions on devices that control the environmental state of the office such as lighting or air conditioning.
- *Project management:* most companies have a dedicated project management service used for planning, issue tracking, and reporting. The interaction with the project management system may prove easier and more natural not by typing, but by speech.

Our system has the following behavior and functionalities:

- Each component of the system that can hold meaningful information about users or means to control the environment should provide voice enabled capabilities to issue commands or request data
- Provides an attendance system
- Requests information and performs actions regarding the ambient ecosystem using voice activated commands
- Performs registration and authentication for the users
- Switches between working modes
- Performs voice activated interaction with the proposed project management tool in a way that it covers the features that come with the software package
- Provides voice activated meeting scheduling and reservation, as well as notification for the actors in the scheduled meeting.

**Figure 1:**
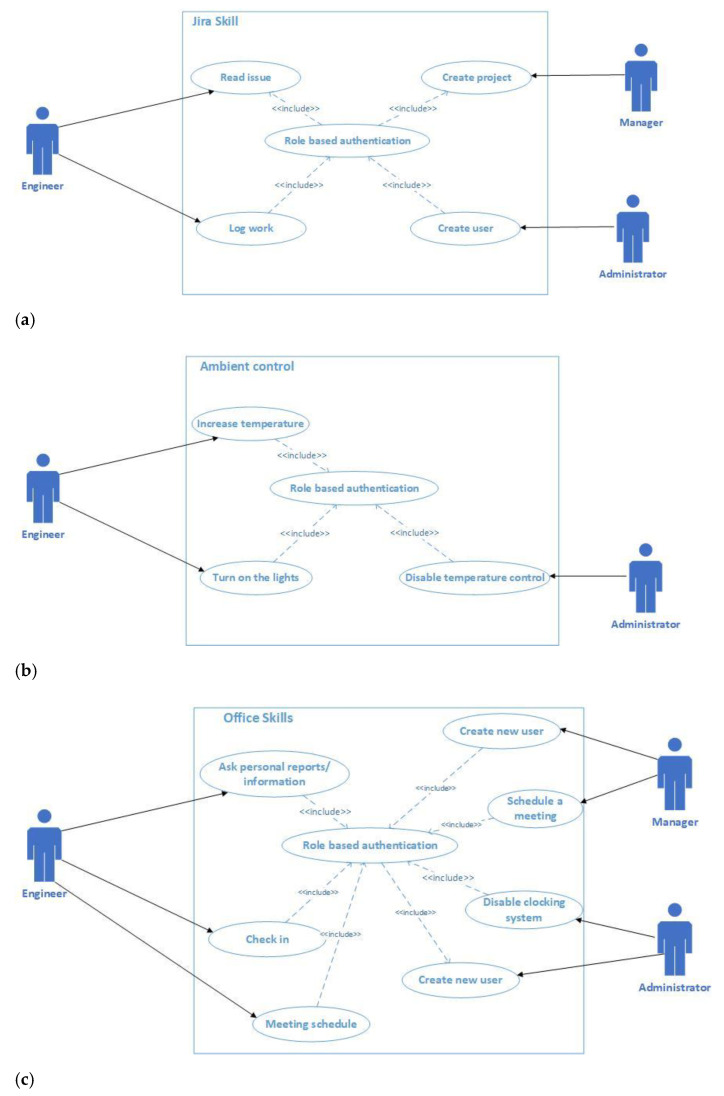
(**a**) Jira skill use case; (**b**) Ambient control use case; (**c**) Office skill use case.

The project management and issue tracking tool we are referring to in our scenario is Jira. Figure 1a presents the Jira voice interaction use case. It involves as actors the Engineer, the Manager, and the Administrator. For all the actors to have access in the Jira Skill, they have to authenticate. After a successful authentication, they can perform different activities, like: the Engineer reads the issue or log to work; the Administrator creates a new user; and the Manager creates a new project.

The Ambient control use case is treated in Figure 1b and the actors are Engineer and Administrator. The two actors for accessing the ambient control have to authenticate, and, based on a successful authentication, will gain access to performing different activities: the Engineer modifies the temperature and turns on/off the lights; and the Administrator may disable the temperature control.

Figure 1c illustrates the voice interaction related to the general office tasks. The actors are Engineer, Manager, and Administrator. For all the actors to have access to the Office skills, they have to successfully pass the authentication process. Based on this, they can make different activities like: Engineer asks for personal reports/information, check in, or the meeting schedule; Administrator may disable the attendance system or create a new user; and Manager can review the employee reports and schedule a meeting.

### 3.1. Proposed Framework

**Figure 2:**
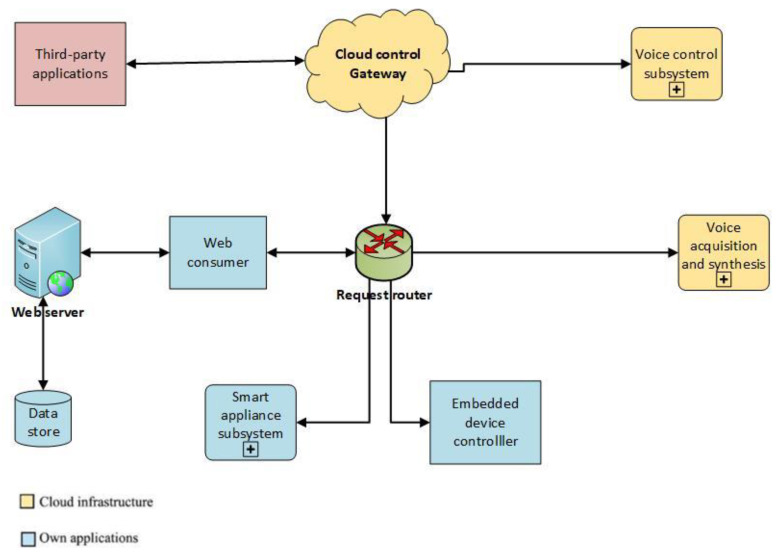
General framework architecture.

Based on the requirements above, the general framework architecture used for our research has been developed. This is based on a multitier architecture, being presented in Figure 2. This can be applied to different smart office scenarios. The services are being separated into different layers, rendering the system more flexible when adding or modifying a specific layer rather than reworking the entire system when the application changes.

The Request Router module will interact with the VA device and will further send the input for obtaining tailored responses. The cloud component is useful for these types of systems because it is always available, and the information needed by the VA can be accessed at any time. The cloud can also collect information from a network of VAs and in this way can improve their functioning. The database will store all the data needed in the system, such as configuration of the IoT hardware, the credentials of the users, and different information required by the VA. The web server will be a broker between the database and the cloud. The cloud will collect the results from the Voice control subsystem, its input coming from the IoT hardware (Smart appliance subsystem and Embedded device controller), and the Voice acquisition and synthesis module. The IoT hardware will have the possibility to communicate with the database and to interrogate the information needed, after the authentication of the party, through the Request Router module. The Web server and the database can be located in the cloud or on a dedicated server; nevertheless, the IoT hardware should be able to access it. The Third-party application module should be customizable for any kind of general application (e.g., project management tools, email management platforms).

The availability of a network connection is a precondition for the Alexa Skills to be accessible since they are hosted on the Amazon Cloud and most of the Alexa Speech Synthesizer is also done on the cloud. In addition, most of the Alexa Skills would include HTTP requests between separated microservices. We can still use some of the features that are related to smart home devices, including switches, lights, and plugs since these devices are directly connected to our local network and subsequently to Alexa. Although our options are limited, the prototype is not constrained by internet availability for basic functions. Since Alexa is using only 256 Kbps for audio streaming, some alternative solutions could be used, like mobile data plans, if redundancy is a crucial aspect in a specific environment.

### 3.2. System Architecture

**Figure 3:**
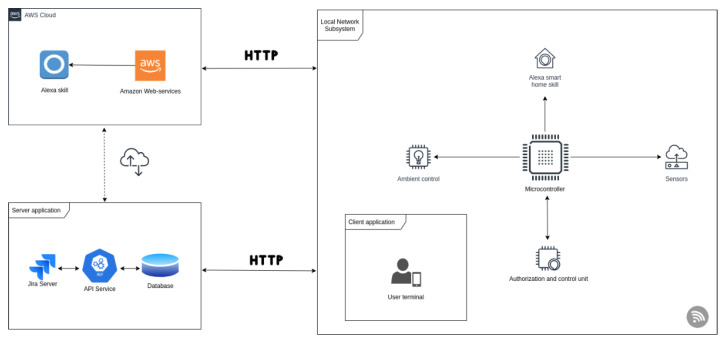
System architecture.

Figure 3 presents the system architecture we implemented for our research. The proposed scenario will integrate in the local network subsystem the following components:

- Raspberry Pi 3 (Sony UK TEC, South Wales, UK) running the Raspbian operating system: is configured for integration with smart appliances such as light bulbs/LEDs, thermostat and other wireless or Bluetooth enabled appliances, but also for integration with microcontrollers such as Arduino or other embedded devices.
- Arduino UNO R3 (Farnell, Romania) connected to embedded components such as the RC522 RFID module.
- DHT11 temperature and humidity sensor (Guangdong, China) for ambient control skills.

Apart from local network subsystem, the following devices are used:

- RC522 RFID (Kuongshun Electronic, Shenzhen, China) module for reading data from RFID/NFC tags, used for attendance and registration system.
- Tenda F3 N300 3 antenna router (Zhengzhou Damulin Electronic, Shenzhen, China) for local network and device discovery.
- TP-link LB120 smart light bulb (Philips Hue, United Kingdom) for the ambient control skill.
- Amazon Echo Dot smart speaker and proprietary Amazon hardware (Amazon, New York, NY, USA) with Alexa Voice Service ready (this we might as well be replaced as with a Raspberry PI, having connected a speaker, a microphone, and Alexa Voice Service, in order to achieve the same result).

The Raspberry Pi and Arduino use serial communication over which they exchange information regarding the state of the RFID reader. The embedded system controller’s main purpose is to collect data from devices that cannot be wireless enabled such as the RFID reader or the humidity and temperature sensor. The smart bulb TP Link LB120 is wireless enabled and discoverable within our network. When the Raspberry Pi receives a socket message coming from the cloud service (in our case, AWS Lambda, as it is noted in Section 3.2.1) and routed through our web server application, it will further propagate the instructions to the smart bulb, ending with the acknowledgement of the updated state.

The following sections will explain in detail the various modules that compose the system architecture implemented for our research.

#### 3.2.1. Software Service Architecture

**Figure 4:**
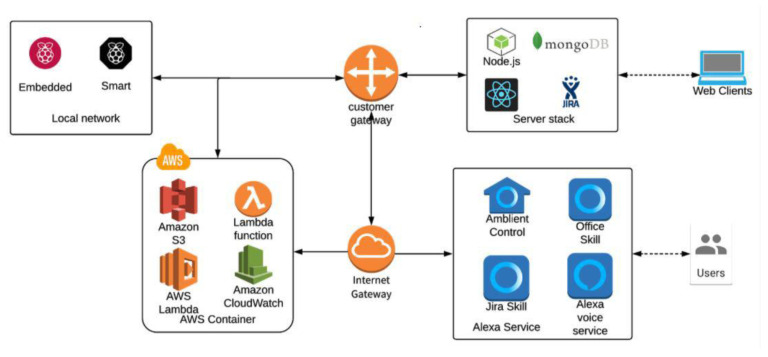
Software service architecture.

Figure 4 presents the software service architecture. An important software module is dedicated to developing the Alexa Service. With the Amazon Alexa platform, natural voice experiences can be built which interact with devices around it. The first component used from Alexa is Alexa Voice Service (AVS) [55], enabling the user’s access to the cloud-based Alexa capabilities with supported hardware kits, software tools, and documentation. It provides the customer basic access to dedicated skills such as asking information about the weather, traffic, news, sport; performing searches on the web; setting alarm clocks; playing music; performing mathematical calculations; online shopping; ordering food, etc. However, more essentially, it allows the developer to install the service on any device (Raspberry Pi, mobile phone, web page) that meets standard requirements (internet, microphone, and speaker). This is an important aspect that is not covered by its competitors which renders Amazon Alexa more accessible to the developers.

Alexa Skill Kit (ASK) [56] enables designers, developers, and brands to build skills tailored to their preference, services, and own devices. It provides its users with dedicated open source standard development kits. Our solution is to use the Alexa Skills Kit SDK for a Node.js platform. In order to develop the tailored skills for our smart office (Ambient Control, Office Skill, and Jira Skill), the building blocks behind voice interaction with a voice assistant are to be used:

- Utterances: include a list of words, phrases, and sentences of what a person might say to Alexa during the interaction in a daily smart office routine. One important aspect of designing a voice experience is defining a wide range of things people may say to fulfill their intent. Basically, the user’s utterance will be mapped to his/her intent.
- Intents: are defined as tasks that a user can ask the new skill to do. This is the entity that will have to capture and map it with the running code for fulfilling the task. As a rule of thumb, it should avoid assuming that the users will utter the words that the developer anticipates. As an example, in our smart office, for the interaction with a light bulb, the defined utterance could be “Alexa ask ambient control to turn on the light”, but the user might say “Alexa ask ambient control to power on the light”.
- Slots: represent variable information in the utterances. In this category, the days of the week, numbers, or any finite state space can be mentioned. This is particularly useful because it allows us to capture slots and use the setting of a certain state of a connected application.
- Wake word: represents the way in which the users tell to the device to start listening because it is about to start a conversation.
- Invocation name: this part of the conversation is used to differentiate between the dedicated Alexa skills and user’s own skill.

The Amazon Web Services (AWS) Container includes the AWS [57] secure cloud services platform. It offers a wide range of affordable service and infrastructure for application based on serverless architecture. The scenario described in this paper can be integrated with Amazon Alexa and third party services. Consequently, it adds an extra layer of security for the interaction between the cloud system and the physical system. AWS Lambda is a serverless computer service [58]. It allows our project to run code in response to events and to automatically manage the underlying computing resources. In AWS Lambda, the triggered events are captured from the configured skills in Alexa Skills Kit, execute subsequent requests to our web service, and issue event responses back to Alexa. It supports the latest updates for the provided programming languages; in our case, it is particularly useful since it offers the latest version of Node.js. Having it coupled with the following service API Gateway, S3 Cloud Storage and CloudWatch will provide a proper environment for deployment code monitoring and logging. Furthermore, it has a flexibility resource model allowing us to allocate the right amount of compute power/function and a convenient pay per use policy. Another important component of AWS Lambda service is that we can do behavioral tests based on different event sources directly in the AWS Lambda tool. This was very useful during the development of the practical application because I would have better traceability than the actual log files, and it reduces the time for manual testing by interacting directly with Alexa Voice Service and receiving feedback from CloudWatch integration.

When interacting with Jira, we will use REST HTTP requests, the authority issuing the request will be our AWS Lambda function. When looking at this flow, an important question arises of how we provide proper authentication and authorization between an AWS Lambda and Jira server both being proprietary software applications and considering the fact that the Jira application server will be installed on our dedicated server and accessible from a public URL.

We use OAuth [59] for integrating Jira Server into the system. Before a user can interact with Alexa skills related to Jira, he must receive an authorization token and provide it to AWS Lambda. These tokens can be further invalidated when we want to opt out of this service. The server will maintain a pool of connected clients (web and devices) that are authorized to interact with the system. The motivation behind using socket connection instead of HTTP is due to the fact that we want real-time feedback for certain actions, as well as having the possibility to manage connections and broadcast messages. Authenticated clients will automatically receive a socket connection to the web server for real-time updates from interacting with Alexa skills.

**Figure 5:**
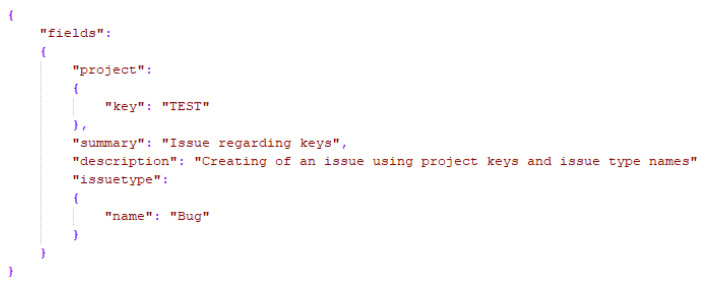
Jira input data.

The input data that we will subsequently send to Jira have to be JSON formatted (Figure 5). This is convenient from a development point of view, since it resembles Javascript object literal syntax and can be used in different programming environments with built-in support. Within an Alexa interaction, we will have to capture slots that are meaningful for the requested skill from a user utterance and consequently format the data in a key-value structure that adheres to the JSON format. Furthermore, these data will be sent to the Jira Server via HTTP on a desired endpoint.

**Figure 6:**
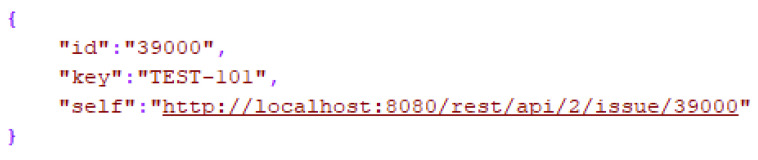
Jira response.

Finally, we receive the response in the expected JSON format (Figure 6). To conclude the interaction with the voice assistant, we can issue a message, build from the response of the requested endpoint, and eventually return a meaningful message back to the user from Alexa.

**Figure 7:**
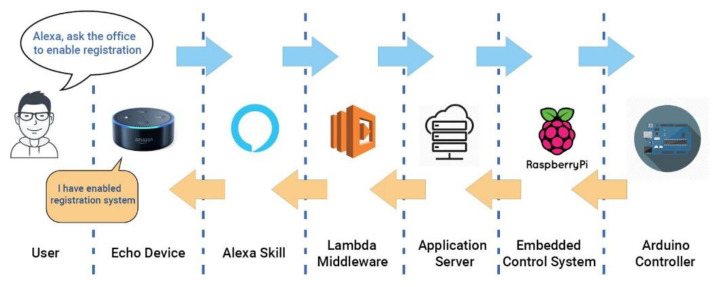
Life cycle of Alexa interaction.

The steps an Alexa interaction will pursue in our system are summarized in Figure 7. It can be noted how an utterance will be transferred to the Alexa Skill module and Lambda Middleware, while the Arduino Controller and Embedded Control System will send back the response message towards the AWS and Echo Device. The interaction with Jira management tool takes place in the input data format and Jira response which were previously presented.

#### 3.2.2. Authorization and Authentication Protocols

When the Lambda Function is authenticated to communicate with the Jira Application Server, the best solution is OAuth, because it describes how distinct servers and services can securely allow authenticated access to their resources without actually disclosing any private credentials.

The Node.js platform is used as a basis for the application server, client, and cloud functions. Part of the final product will be a web interface where users can authenticate, monitor real-time interaction with smart appliances controlled by Alexa in their proximity and check their status in the company (attendance system). Thus, we require some basic authentication for the web client. The solution in this case is Passport [60], which is a middleware for Node.js. It also values encapsulation of the components and maintainable code. Moreover, it is extendable in a manner where the authentication could be extended to multiple login strategies—for example, login with Google, Facebook, or Twitter. We propose to use JSON Web Tokens to authenticate and authorize devices and web clients for more sensitive information exchange such as event triggers for smart appliances. The information can be verified and trusted because it is digitally signed. In this way, the tokens can be signed using a secret or a public/private key pair using RSA or ECDSA.

#### 3.2.3. Web Server

The dedicated server hosting has 4 GB RAM memory, 25 GB SSD Disk and 2 vCPUs and is running Ubuntu 16.04.4 × 64. Here, we will host the services necessary for such a system that are not related with the interaction with Alexa Skills Kit, but consists of data and event source that provide feedback to the voice assistant.

The server application is built using a Node.js run-time environment and, more precisely, the implementation is done using the Express.js framework. This framework allows us to setup middleware to respond to HTTP requests, defining a routing table used to perform different actions using HTTP methods and URLs, and it will dynamically serve and render our web client application. The Express application will export a public port where the users will connect; consequently, we will use Nginx web server as a reverse DNS proxy for our application, and we will define corresponding server blocks to map our applications to the associated DNS records.

The web client application refers to the visual component as part of the human–computer interaction model. It is implemented in React [61] and will be a web page where administrators can add new users to the system or monitor the status of the users or connected devices. As data store is part of our system, MongoDB is being used, which is a document database that stores data in a flexible document format. This means that fields can vary from document to document and data structure can be changed over time. In future iterations of this project, it is expected that new devices and new skills can be easily added to the system so this type of permissive structuring can be used in this case.

## 4. Results

This section is implementing steps 4, 5, 6, and 7 from the research methodology presented in Section 3. Firstly, we investigate the user perception when interacting with the prototype. For this, different test cases have been created. The results of a feedback survey express the degree in which the user is interacting with the system. Validation applies principles of affective computing. Furthermore, the task completion factor is used to better understand the causes for users’ choices on the preferred customized Amazon Alexa skill.

### 4.1. Usability Evaluation

Once the system was built and the skills were completely developed, our first step was to conduct a usability evaluation in order to understand the impact that the system and the skills could have on the smart office users. Our aim was to measure the acceptance level of the three main developed skills, by understanding which skill would be mostly used in the future interaction of the users. The measurements will allow us to determine if the skills have a positive or negative impact over the user and to determine which features need further adjustments and investigation.

**Figure 8:**
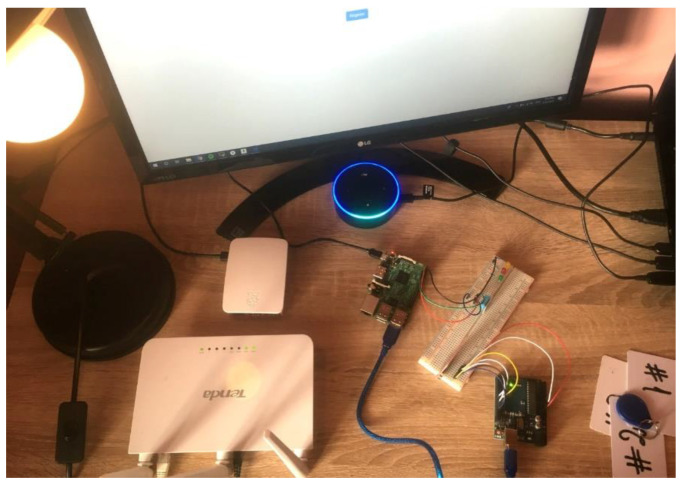
Experimental setup.

The evaluation was conducted over the period of three months, from 1 August until 1 November 2020, in a partnership that the university had with a local IT company. The total number of subjects participating in the evaluation was 71 employees and students. The idea of the project was presented via online meetings to 43 students from the 3rd year of study in Computer Science, as well as to 28 employees of the company. The system was deployed on a testing station at the company’s headquarters, this testing station being able to be remotely accessed. The office is an open-space environment; therefore, different noises could be produced while testing. This aspect can influence the results of the experiments. The experimental setup can be noticed in Figure 8. The users were trained in order to ensure that the user experience is aligned with the voice design principles, but also the interactions adhere to using the right intent schema, utterances, and custom slot types.

The system behavior was analyzed in the context of end-to-end interactions with the voice assistant in different smart office scenarios. We will avoid confusing the system on purpose because it will render our results irrelevant since the working principle of Alexa Voice Service is matching dictionary words based on confidence levels rather than speech to text translation. We designed our test-cases on three different categories based on the complexity of the utterances. The selected test cases are shown in Table 1, where difficulty represents the complexity of the utterance, and response is the expected output from Alexa.

**Figure 9:**
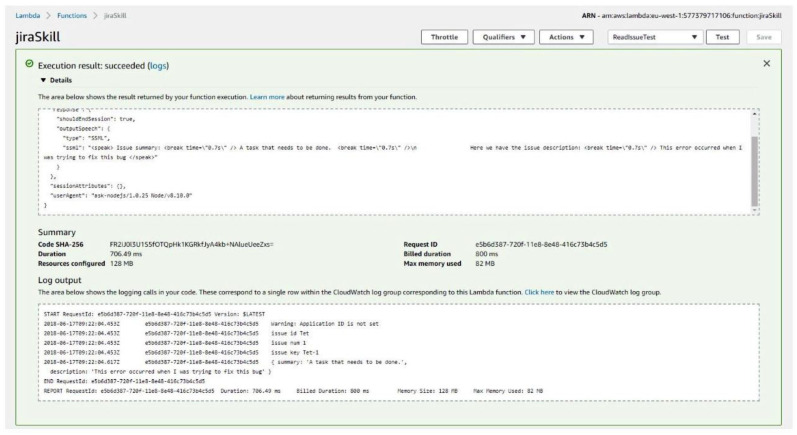
Successful execution of the system.

In Figure 9, we are considering, as an example, the following interaction between the user and our voice assistant. Given a list of possible utterances required by the voice assistant to activate a skill (in our case the skill performs the reading of a JIRA ticket for a given project ID and a number), the user says: *“Alexa, read issue {issueID} {issueNumber}”*; subsequently, the voice assistant maps his utterances to the corresponding skill named *JiraReadIssueSkill*, as well as extracting the requested variables *issueID*, *issueNumber*. We can observe under Log Output the workflow: the system identifies that the user uttered the *issueID* = *TET*, and *issueNumber* = 1, building the *issueKEY* = *TET*-1 which corresponds to the JIRA ticket in our database, we get back as a response a JSON object having a summary and a description which corresponds to the ticket. The JSON response is then used by our voice assistant to provide us the information we requested. As seen under the Details tab, the system builds a reply to the user using Speech Synthesis Markup Language (SSML). After providing feedback to the user, the communication session is ended and the voice assistant waits again for the wake word.

**Figure 10:**
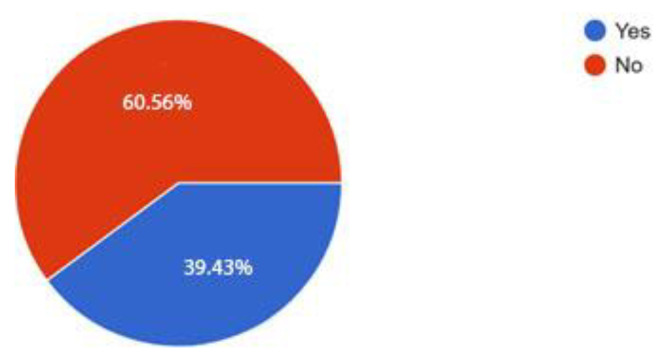
Obtained statistical data for the first question in the initial survey.

**Figure 11:**
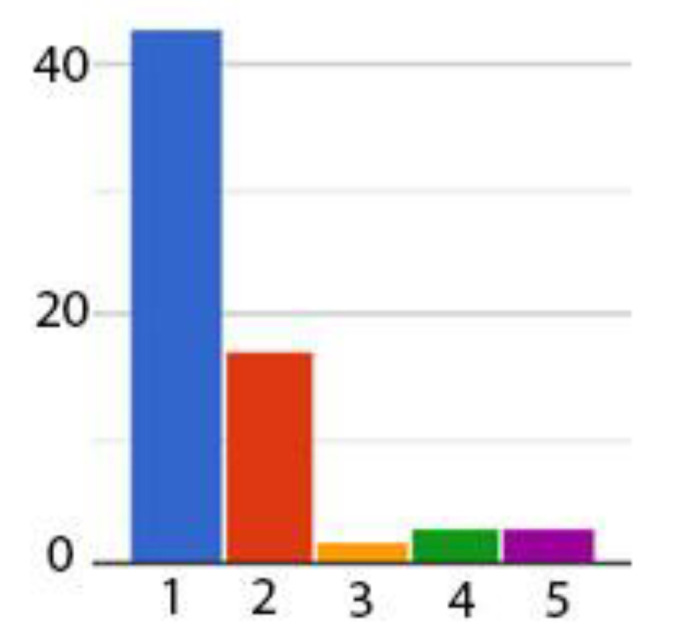
Obtained statistical data for the second question in the initial survey.

We have followed the state-of-the-art literature in VAs which presents the methodology for usability evaluation [41,54]. Therefore, at the beginning of the research, we have applied an initial survey in order to determine if the participants were already familiar with Amazon Alexa technology. Out of the 71 participants, 51 (71.8%) were male and 20 (28.2%) were female. The age distribution of the group is presented in Table 2. The first question in the survey (Table 3) aimed at understanding if our users have previously used Amazon Alexa. The results show that 60.56% users have never used this technology (Figure 10). The results from the second question show that a total of four users are using a VA device weekly or several times per week and only one is using it on a daily basis (in Figure 11, the question’s number is represented on the x-coordinate and the number of respondents is represented on the y-coordinate).

**Figure 12:**
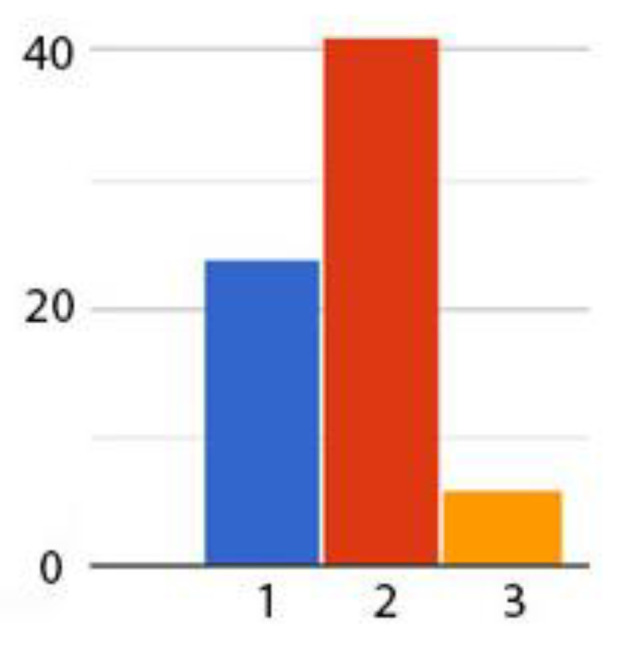
Users’ preferences for the skills.

The next step after users’ interaction with the three types of skills was to design a survey (Table 4) that the users filled out at the end of their interaction with Amazon Alexa skills. We were interested in the personal experience of the users, this being the reason for which the first question aims at understanding which of the three skills was the one to be mostly used in the future. The results reveal that 24 of the respondents prefer the Office skill, 41 favor the Ambient control skill and only 6 are for the Jira skill (Figure 12). The question on additional smart office skills reveal that the users would be interested in different skills like: “improved light control”, “elderly people features”, “specific song requests”, “volume control”, “microphone control”, “Integrated Development Environment control”, “connection to additional devices”.

The last question is an open answer question because we wanted to validate the results from the first question. We gathered answers at this question from 29 users. The validation process would use sentiment analysis tools applied on the corpus of text received from the users as answers to the last question. This will reveal the polarity of the users for the three main skills. We decided to analyze our corpus with Lexalytics [62] and SentiStrength [63] tools.

In Table 5 the polarity results obtained on the corpus from the last question are being presented. For the Office skill the results from Lexalytics are positive-neutral as the final score is +0.167. This is partially in accordance with the result obtained from SentiStrength because the score is 3 (5 is marked by SentiStrength as extremely positive and 1 as not positive). For the Ambient control skill the results from Lexalytics are positive, this being the result from SentiStrength also. Regarding the Jira skill, the results from Lexalytics are negative-neutral and those from SentiStrength show that are “not positive.”

### 4.2. Task Completion

The usability evaluation showed that the Jira skill had the lowest sentiment analysis score. We wanted to further investigate the causes for this score. Starting with this goal, the next step was to compute the task completion factor, which is measuring the task success probability of dialogue corpora [25]. For this, we used the PARADISE (PARAdigm for DIalogue System Evaluation) framework [25,64]. This framework uses the Kappa coefficient, k, to functionalize the measure of task-based operation success. The computation of the Kappa coefficient is based on independently distributed confusion matrix, as presented in Table 6. According to [64,65] the diagonal and off-diagonal values are to be filled-out with the correctly and incorrectly identified utterances from the three skills scenarios.

The computation of the Kappa coefficient is presented in Equation (1).

(1) $$k=\frac{P\left(A\right)-P\left(B\right)}{1-P\left(E\right)}$$

*P*(*A*) is the proportion of times that agreement occurs between the actual set of utterances in the implemented skill with the scenario attribute value and can be calculate from the confusion matrix according to Equation (2) and is named the Actual Agreement [25,64].

(2) $$P\left(A\right)=\frac{\sum_{i=1}^{n}M\left(i,i\right)}{T}$$

The sum of frequency *t*_1_ + *t*_2_ + … + *t_n_* in the confusion matrix is calculated as *T*. *P*(*E*) can be calculated according to Equation (3), being named the Expected Agreement. In this equation the sum of the *i*th column frequency of the confusion matrix is noted as *t_i_*.

(3) $$P\left(E\right)=\overset{n}{\underset{i=1}{\sum }}{\left(\frac{t_{i}}{T}\right)}^{2}$$

The task completion computation based on the PARADISE framework gives the Actual Agreement *P*(*A*) = 0.770 and the Expected Agreement *P*(*E*) = 0.367. The Kappa coefficient can be calculated according to Equation (1) and gives the value *k* = 0.636. According to [64], the interpretation of this score shows that our Alexa-based system is ‘Substantial.’ This can be understood from the confusion matrix where the Jira skills has the highest number of missed utterances.

### 4.3. Results’ Discussion

The (RQ2) of our paper is meant to understand which are the most accepted tasks to be performed with the help of voice assistants in a smart office environment. The initial survey has resembling results with other state-of-the-art papers [41,54], namely Amazon Alexa is seldom used by the users during a given amount of time, like one week. The survey applied at the end of the interaction test cases are meant to reveal this information. The first question of the survey, “Which of the three skills you are most probably to use again”, offers a first insight into answering (RQ2). It shows that the most appreciated skill is the Ambient control and the least desired skill is Jira. It is interesting to notice that the Ambient control is a moderate complexity skill, while Jira is the most difficult one. The practical scenarios show that Alexa has difficulties in understanding the project’s name and the user name. This is the case in other research papers as well, especially in the medical field [29,53]. In this point, we wanted to further understand which is the users’ perception of the implemented Alexa skills, in this way answering (RQ3). This was the reason that the last question offered an open answer. The sentiment analysis we applied on the formed corpus maintains the idea that the Jira skill has a negative-neutral polarity. The task completion computation reveals that the cause for the users’ lack of satisfaction stays in the incapacity of Jira skill to complete the tasks at hand. This comes to sustain the conclusion of the first question and reveals future research directions, like the optimization of the utterances construction.

The slots in the tested utterances can only be defined as a finite set of values that we have to manually add to the system. The reason behind this design decision is fair since is a shared service and users might be tempted to overload the system for malicious intents. The utterances in this system have to be valid in Standard English (or one of the available languages) dictionary words. This is a showstopper and a possible hazard in the system if the user starts to use codes, passwords, keys, or slang. Further research is needed in this direction, but that is not the scope of our paper.

The intention of the developers was zero configuration integration for smart appliances. However, some setup is required to provide secure means of communication. Other issues which can appear in the described system are speech impediments, blocking of the microphone, noise, or disability (not possible to solve, so contingency plans are recommended).

## 5. Conclusions

This paper is presenting a solution which integrates a voice assistant in a smart office scenario, as a response to our (RQ1). Our particular interest was to develop the environment to interact with specific smart office devices, as well as to project management tools, by this offering new scientific insights that can guide researchers on future, similar work. For addressing (RQ1) we have developed the integration in an IT testing environment which was successful, but also three different Alexa skills: Office, Ambient control and Jira skills. In order to gather insights regarding the usage of each skills, we have carried out a usability evaluation, based on the state-of-the-art literature review. This showed us that most of the users have not previously used VAs and even those who are using these devices, are using it very rarely. This step gave us a better insight into understanding the received responses at (RQ2) and (RQ3).

In order to address (RQ2) we have developed a feedback survey that revealed that Jira skill is the least appreciated skill. In other words, the system is stable especially for easy and moderate utterances, regardless of the user’s experience. A further, deeper analysis (RQ3) showed that the cause for the user dissatisfaction is the incapacity of the system to understand complex utterances. The task completion investigation confirmed this result for obtaining a ‘Substantial’ overall score for the prototype. This is the reason that further optimizations are needed in constructing complex inputs for Alexa.

As future work, we plan to increase the control on how Alexa is responding, by taking into consideration the fact that user might be pausing for longer periods of time or even would like to express excitement. As part of natural language processing we want to build models that can predict and classify intents and utterances based on unstructured inputs mispronunciation, contradiction and swapped words.

## Figures and Tables

**Table 1:** Experimental test cases.

Skill	Difficulty	Utterance	Response
Office	Easy	Ask the office who is on online?	A list of online users
Ambient control	Moderate	Tell ambient control to turn {on/off} the light	Notice to update
Jira	Hard	Ask Jira to create a {software/business} project {* any name} and assign {* any username} as manager	Confirmation notice of the request

**Table 2:** Age distribution of the users.

Age	Number	Distribution
20–25	42	59.15%
26–30	15	21.12%
31–35	9	12.67%
36–40	2	2.81%
>40	3	4.22%

**Table 3:** Initial survey questions (adapted after [41,54]).

Question	Possible Answers
Have you previously used voice-activated devices, like Amazon Alexa?	Yes No
If you have previously used, how often do you still use those devices?	Never Seldom Daily Weekly Several times per week

**Table 4:** Feedback survey questions (adapted after [41,54]).

Question	Possible Answers
Which of the three skills you are most probably to use again	Office skill Ambient control skill Jira skill
Do you like to receive notifications through Alexa-enabled devices?	Yes No
What additional smart office skills would you like to use through the Alexa?	Open Answer
Please describe you experience with the three tested skills	Open Answer

**Table 5:** Polarity results for the last question of the survey, using different tools.

Skill	Tool	Polarity
Office	Lexalytics Sentistrength	Neutral (+0.167) positive strength 3 and negative strength −1
Ambient control	Lexalytics Sentistrength	Positive (+0.790) positive strength 3 and negative strength −1
Jira	Lexalytics Sentistrength	Neutral (−0.047) positive strength 1 and negative strength −1

**Table 6:** Confusion matrix for the three scenarios.

	Office Skill	Ambient Control Skill	Jira Skill
Office skill	26	2	1
Ambient control skill	1	27	1
Jira skill	7	8	14

## Data Availability

Data sharing not applicable.
